# Cross-contamination by disinfectant towelettes varies by product chemistry and strain

**DOI:** 10.1186/s13756-020-00797-4

**Published:** 2020-08-24

**Authors:** Maxwell G. Voorn, Summer E. Goss, Carine A. Nkemngong, Xiaobao Li, Peter J. Teska, Haley F. Oliver

**Affiliations:** 1 Department of Food Science, Purdue University, 745 Agriculture Mall Drive, West Lafayette, IN 47907 USA; 2 Diversey Inc., Charlotte, NC 28273 USA

**Keywords:** *Staphylococcus aureus*, *Pseudomonas aeruginosa*, Disinfectant towelettes, Bactericidal efficacy, Cross-contamination

## Abstract

**Background:**

Disinfectant products are used frequently on environmental surfaces (e.g. medical equipment, countertops, patient beds) and patient care equipment within healthcare facilities. The purpose of this study was to assess the risk of cross-contamination of *Staphylococcus aureus* and *Pseudomonas aeruginosa* during and after disinfection of predetermined surface areas with ready-to-use (RTU) pre-wetted disinfectant towelettes.

**Methods:**

This study tested six disinfectant towelette products against *S. aureus* ATCC CRM-6538 and *P. aeruginosa* strain ATCC-15442 on Formica surfaces. Each disinfectant was evaluated on a hard nonporous surface and efficacy was measured every 0.5 m^2^ using a modified version of EPA MLB SOP-MB-33 to study the risk of cross-contamination.

**Results:**

We found that all of the wipes used in this study transferred *S. aureus* and *P. aeruginosa* from an inoculated surface to previously uncontaminated surfaces. Disinfectant towelettes with certain chemistries also retained a high level of viable bacteria after disinfection of the surface area. The cross-contamination risk also varied by product chemistry and bacterial strain.

**Conclusion:**

Disinfectant wipes can cross-contaminate hard nonporous surfaces and retain viable bacterial cells post-disinfection, especially over larger surface areas. This highlights a need to further investigate the risk disinfectant wipes pose during and post-disinfection and guidance on maximum surface areas treated with a single towelette.

## Background

Healthcare Acquired Infections (HAIs) are prevalent in healthcare settings and are becoming harder to treat especially as levels of multidrug resistant infections are on the rise [1]. According to the Center for Disease Control and Prevention (CDC), approximately one in 31 United States (US) patients will contract at least one HAI within 4 days of health care facility admission [2]. Although this is an improvement from 2011 statistics with daily HAI incidence rates of at least one in 25 patients, in 2015, an estimated 633,300 US patients suffered from 687,200 HAI [3]. The most prevalent infection rates occur in acute care hospitals (ACHs), predominantly due to opportunistic pathogens occurring in healthcare settings [4], and the HAI tracking and reporting requirements for acute care facilities to the National Healthcare Safety Network of the CDC. The risk of a HAI occurring is highest among immunocompromised individuals [5]; with mortality rates of approximately 11% among hospitalized patients suffering from HAIs [3].

*Staphylococcus aureus* and *P. aeruginosa* are among the most prevalent etiological agents of HAIs [6]. *S. aureus* is typically harmless to a healthy individual, but can cause deadly infections, such as septicemia, endocarditis, osteoarticular infections, and pleuropulmonary infections [7]. *P. aeruginosa* can also cause infections such as cystic fibrosis [8] septicemia and pneumonia that can be fatal for immunocompromised individuals [9].

Hard nonporous environmental surfaces in healthcare facilities harbor pathogens that cause HAIs [10]. *S. aureus* and *P. aeruginosa* have been detected from bedside cupboards, bed rails, floors and other hospital equipment [11]. *S. aureus* and *P. aeruginosa* persist on these surfaces [12, 13], increasing transmission risk resulting in HAIs [14]. Overall, pathogen persistence could be due to sub-lethal concentrations of disinfectants [15, 16], low efficacy levels for some classes of disinfectant wipes [17, 18], amount of surface area wiped [18], and the label-use recommendations not being followed [19].

Healthcare personnel rely on disinfectant wipes for environmental surface disinfection [20, 21]. Previous studies have focused mainly on the bactericidal efficacy of disinfectants under label and off-label use conditions [20, 22]. However, despite widespread use of disinfectants, limited studies [23–25] have evaluated the risk of disinfectant towelettes cross-contaminating previously uncontaminated surfaces during the wiping process from an inoculum source. The objectives of this study were to (i) evaluate the risk of disinfectant towelettes transferring *S. aureus* or *P. aeruginosa* from an inoculated zone to otherwise not contaminated surfaces and (ii) detect levels of *S. aureus* or *P. aeruginosa* on disinfectant towelettes after the wiping process. We hypothesized that during the wiping process, disinfectant towelettes are capable of transferring pathogens to otherwise low-risk areas of an environmental surface. We also hypothesized that post-disinfection, and following label-defined contact times, towelettes may remain contaminated with viable *S. aureus* or *P. aeruginosa*.

## Methods

### Bacteria, disinfectant towelettes, and surface type used for testing

**Fig. 1:**
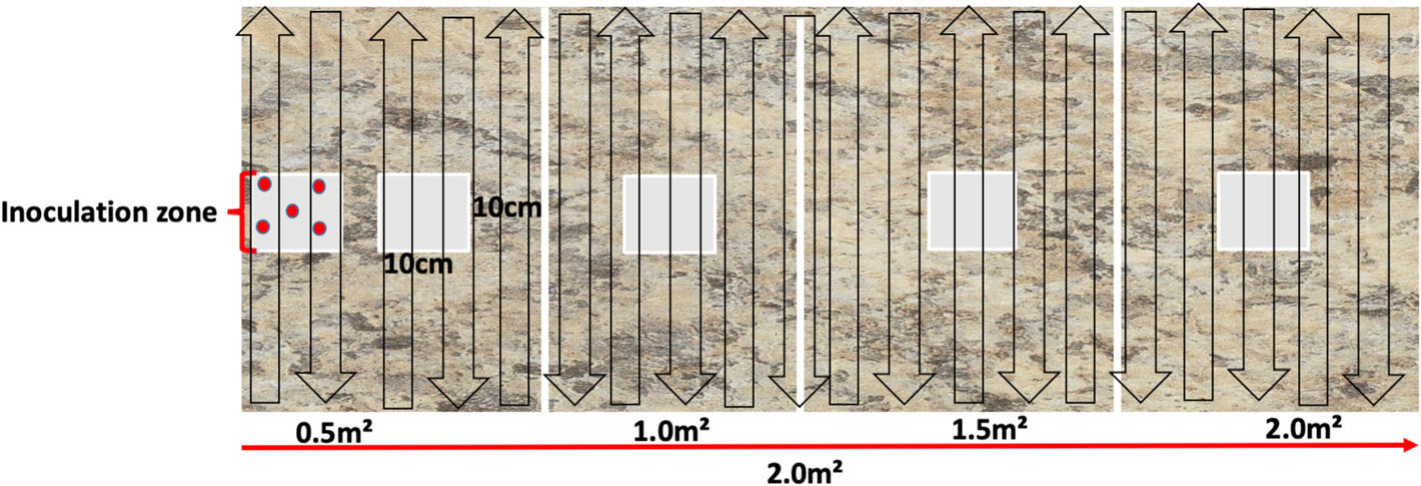
Schematic diagram of the Formica surface used for wipe testing. Two meters of Formica were delineated into 0.5 m^2^ sections. Approximately 2.5 × 10^7^ log_10_ CFU of *S. aureus* or *P. aeruginosa* were spotted onto the i-zone (red dots). The surface was wiped in an up and down pattern as indicated by black outlined arrows from left to right. Light grey squares 10 cm × 10 cm (100 cm^2^) were sampled to recover potentially cross-contaminated *S. aureus* or *P. aeruginosa*

This study tested six disinfectant towelettes (Table 1) that are commonly used in healthcare facilities for equipment and environmental surface disinfection. The experiment proceeded with Formica (laminate) imitation-granite surface, which was two square meters in length as previously described by Nkemngong et al., 2020 (submitted). Briefly, the Formica board was partitioned into five testing zones: the inoculation zone (i-zone) and one-half (0.5 m^2^), one (1.0 m^2^), one and a half (1.5 m^2^), and two square meters (2.0 m^2^) from the i-zone (Fig. 1). The zones for testing (swabbing) were 10 × 10 cm (0.01 m^2^) in size. The disinfectant towelette itself was also analyzed after the wiping procedure was complete.

**Table 1:** Active ingredients and contact times for disinfectant wipes tested in this study

Disinfectant product ^a^	Disinfectant Active Ingredient(s)^b,c^	Active level at use^d^	Label contact time (mins)^f^
HP1	1.4% hydrogen peroxide	1.4%	1
HP2	0.5% hydrogen peroxide	0.5%	1
HP3	0.5% hydrogen peroxide	0.5%	1
QA1	0.25% n-alkyl (68%C_12_, 32%C_14_) dimethylethylbenzyl ammonium chloride 0.25% n-alkyl (60% C_14_, 30% C_16_, 5% C_12_, 5% C_18_) dimethyl benzyl ammonium chloride 55% isopropanol	0.5% + 55% ^e^	2
QA2	0.76% didecyldimethyl ammonium chloride 15% isopropanol 7.50% ethanol	0.76% + 22.5%^e^	1
QA3	0.233% disobutylphenolxyethoxyethyl dimethyl benzyl ammonium chloride 14.3% isopropanol	0.233% + 14.3% ^e^	2

^a^Abbreviated naming scheme reflects aggregated active ingredients for commercially available EPA registered disinfectants used in this study;

^b^Active ingredient concentration;

^c^Dilution at use, ready-to-use;

^d^Active ingredients concentration;

^e^Total quaternary ammonium plus alcohol content;

^f^Defined label contact time

### Surface preparation, wiping method, and sample collection

The Formica was cleaned and disinfected between trials as previously described by Nkemngong et al., 2020. Briefly, the surface was wetted with a 10% bleach solution, followed by rinsing with sterile deionized water. It was then followed by a standard house-hold neutral agent, containing 0.05% Thymol, to reduce chemical residue on the prepared surface. The neutralizing agent was allowed to sit for its recommended contact time (two minutes) before following with a second rinse with sterile deionized water. Lastly, before surface inoculation, 70% ethanol was applied to the Formica sheet and allowed to air dry.

A total of 50 μL of approximately 5.0 × 10^8^ log_10_ CFU/ml in 10 μL aliquots (2.5 × 10^7^ CFU) were dispensed unto the i-zone, and the wiping procedure followed a modified version of the EPA SOP MB-33-00 for *S. aureus* and *P. aeruginosa* [26]. Once inoculated, the culture was allowed to air-dry before using the towelette to wipe the surface from the i-zone to the 2.0 m^2^ mark (Fig. 1). From each RTU disinfectant product, the first two towelettes were discarded and the third towelette was used for testing, to ensure it was fully loaded with disinfectant liquid. The wiping process began at the bottom left corner of the Formica board (below where the board was inoculated) and was wiped evenly in an up-down pattern with consistent speed and pressure from the i-zone to the 2.0 m^2^ mark. Once the wiping procedure was complete, the disinfectant was left undisturbed for its label-defined contact time (Table 1). At the end of the contact time, the towelette itself was placed in a sterile stomacher bag with 50 mL of 0.52% neutralizing buffer (BD Difco, Becton, Dickinson and Company, MD, USA). Swab samples were also collected from standard 10 cm × 10 cm (100 cm^2^) sampling areas within each partitioned zone (i-zone, 0.5m^2^, 1.0 m^2^, 1.5m^2^ and 2.0m^2^). Samples for *S. aureus* or *P. aeruginosa* detection were collected using PUR-Blue Swabs (World BioProducts, Libertyville, IL; with 10 mL sterile HiCap neutralizing buffer).

### Pathogen detection and enumeration

After the wiping procedure and sample collection, each swab sampler was vortexed for 30s to release bacteria cells from the sponge of the swab sampler into 10 mL sterile neutralizing buffer (World BioProducts, Libertyville, IL). Used towelettes in 50 mL of 0.52% neutralizing buffer (BD Difco, Becton, Dickinson and Company, MD, USA) were stomached at 200 rpm for 5 min to release bacteria cells trapped on the towelettes into the neutralizing buffer. Ten mL aliquots from swab samplers and wipe samples were vacuum-filtered onto sterile filter membranes (0.2 μm pore; Pall Corporation, Port Washington, NY) and plated on tryptic soy agar (TSA; BD Biosciences, San Jose, CA) for *S. aureus* and Reasoner’s 2A agar (R2a; Becton, Dickinson and Company Sparks, MD) for *P. aeruginosa*. Plates were incubated at 37 °C for 24 ± 2 h prior to counting colony forming units (CFU).

### Statistical analyses

*S. aureus* and *P. aeruginosa* were recovered after wiping five separate test zones of a two-meter square Formica sheet and recovered CFU were log_10_ -transformed. The disinfectant towelette was also tested for viable *S. aureus* and *P. aeruginosa* CFU post-disinfection of the two-meter square surface area. Average log_10_ CFU loads were calculated for towelettes and sampled surface areas, which were used to test for statistically significant differences among six disinfectant products. The least squares method of the Proc Glimmix test was used to fit linear models (*n* = 36, α = 0.05) and interactions amongst disinfectant products, sampled surfaces, and log_10_ densities on towelettes. Treating both the product type and surface area as continuous variables throughout the data analysis, Tukey adjustments were used to analyze statistically significant differences between mean log_10_ CFU/100 cm^2^ counts recovered post-disinfection on surface areas treated. The same procedure was used to test for significant differences among average log_10_ CFU on used disinfectant towelettes. All statistical tests were conducted using SAS version 9.4 (SAS institute, Cary, NC).

## Results

### *S. aureus* detected on previously uncontaminated surfaces post-disinfection with towelettes

**Fig. 2:**
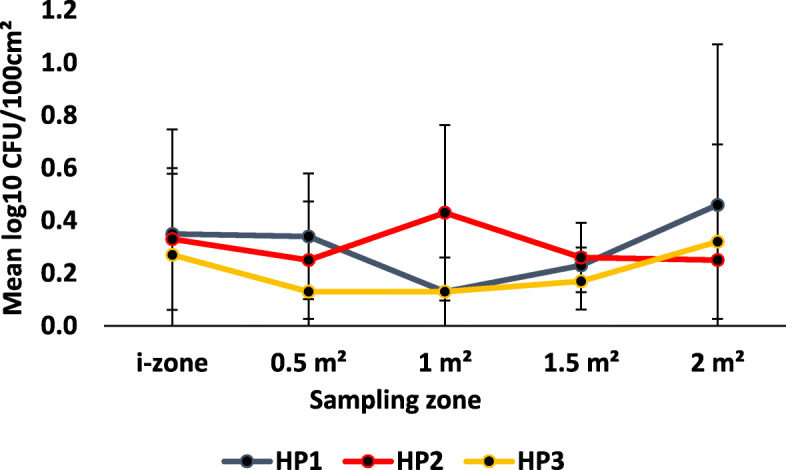
Mean log_10_ CFU/100 cm^2^ remaining on sampled portions of the Formica sheet post-disinfection with hydrogen peroxide disinfectant towelettes challenged with *S. aureus*

**Fig. 3:**
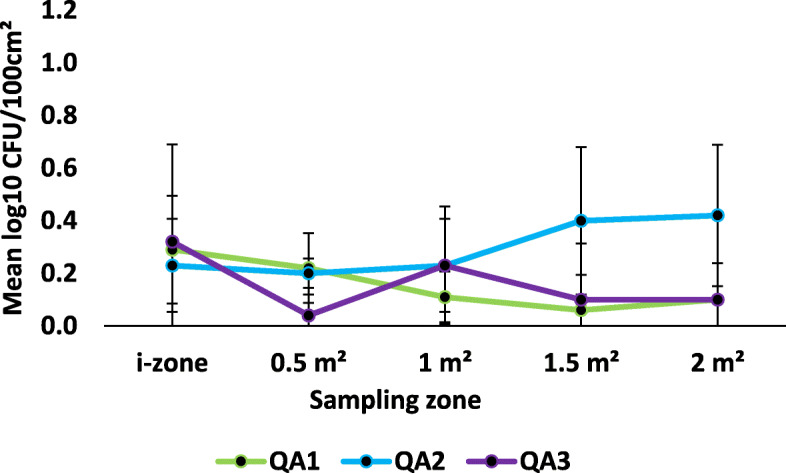
Mean log_10_ CFU/100 cm^2^ remaining on sampled portions of the Formica sheet post-disinfection with quaternary ammonium alcohol disinfectant towelettes challenged with *S. aureus*

Irrespective of disinfectant product, ready-to-use disinfectant towelettes transferred *S. aureus* to previously uncontaminated surfaces from the i-zone (Figs. 2 & 3). Overall, the towelettes transferred on average, 0.19 ± 0.18 and 0.21 ± 0.21 log_10_ CFU/100 cm^2^ to the 0.5 m^2^ and 1.0 m^2^ surface areas, respectively. Disinfectant towelettes also transferred a mean of 0.20 ± 0.19 and 0.27 ± 0.34 log_10_ CFU/100 cm^2^ from the i-zone to the 1.5 m^2^ and 2.0 m^2^ surface areas regardless of the product being tested.

Regardless of the sampling zone, there were no statistically significant differences among the log_10_ CFU/100 cm^2^ detected from previously uncontaminated surfaces when wipes were challenged with *S. aureus* (*P* > 0.3499; Figs. 2 & 3). The product type was also not statistically relevant overall (*P* > 0.0756). Specifically, none of the products transferred statistically significant different log_10_ CFU/100 cm^2^ to the 0.5 m^2^, 1.0 m^2^, 1.5 m^2^ and 2.0 m^2^ compared to the log_10_ CFU/100 cm^2^ recovered from the i- zone post-disinfection (*P* > 0.05; Figs. 2 & 3).

### Varying levels of *P. aeruginosa* were transferred by disinfectant wipes to uncontaminated surfaces

**Fig. 4:**
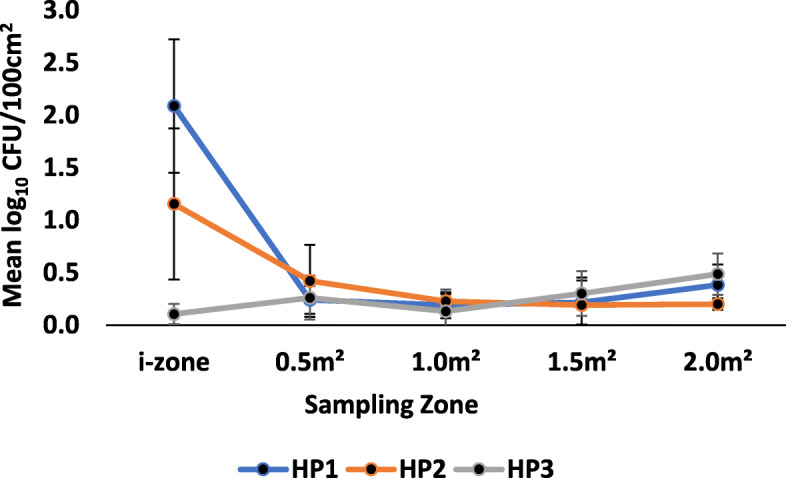
Mean log_10_ CFU/100 cm^2^ remaining on sampled portions of the Formica sheet post-disinfection with hydrogen peroxide disinfectant towelettes challenged with *P. aeruginosa*

**Fig. 5:**
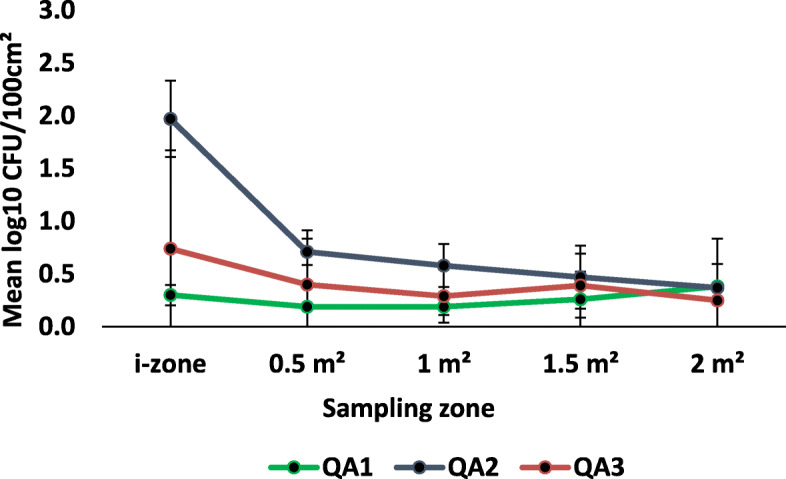
Mean log_10_ CFU/100 cm^2^ remaining on sampled portions of the Formica sheet post-disinfection with quaternary ammonium alcohol disinfectant towelettes challenged with *P. aeruginosa*

*P. aeruginosa* was transferred from the i-zone to previously uncontaminated low-risk surfaces by ready-to-use disinfectant towelettes (Figs. 4 & 5). When transfer levels for all wipes were averaged, a mean of 0.37 ± 0.33 log_10_ CFU/100 cm^2^ and 0.27 ± 0.23 log_10_ CFU/100 cm^2^ were transferred from the i-zone to the 0.5 m^2^ and 1.0 m^2^ surface areas respectively. From the i-zone to the 1.5 m^2^ and 2.0 m^2^ surface areas, each towelette transferred an average of 0.31 ± 0.26 and 0.35 ± 0.27 log_10_ CFU/100 cm^2^, respectively, onto hard nonporous low risk surfaces regardless of the product type.

Regardless of the sampling zone, the surface area wiped was statistically significant, and there were relevant differences between the i-zone and uncontaminated surfaces (*P* < 0.0001; Figs. 4 & 5 . Irrespective of product type, the average log_10_ CFU/100 cm^2^ transferred to the 0.5 m^2^, 1.0 m^2^, 1.5 m^2^ and 2.0 m^2^ surface areas were significantly lower than the log_10_ CFU/100 cm^2^ recovered from the i- zone after surface disinfection (*P* < 0.05). There were no statistically significant differences in the mean log_10_ CFU/100 cm^2^ between the 0.5 m^2^, 1.0 m^2^, 1.5 m^2^ and 2.0 m^2^ uncontaminated surface areas (*P* ≥ 0.05; Figs. 4 & 5).

Product type was statistically relevant (*P* < 0.0001; Figs. 3 & 4). Within the i-zone, QA1 and QA3 had a significantly lower log_10_ reduction than QA2 (*P* < 0.05; Fig. 5). Specifically, QA2 transferred significantly higher log_10_ CFU/100 cm^2^ from the i-zone to the uncontaminated surface areas (0.5 m^2^, 1.0 m^2^, 1.5 m^2^, and 2.0 m^2^) than QA1 or QA3 (*P* < 0.05; Fig. 5). Within the i-zone, HP2 and HP3 were not statistically significantly different ((*P* ≥ 0.05; Fig. 4). However, HP3 had a significantly higher log_10_ reduction (lower log_10_ CFU/100 cm^2^) compared to HP1 within the i-zone (*P* < 0.05; Fig. 4). HP2 and HP3 were also significantly lower when compared to disinfectant wipe QA2 (*P* < 0.05; Figs. 4 & 5). There was, however, no statistically significant difference between QA2 and HP1 (*P* ≥ 0.05; Figs. 4 & 5). There were also no statistically significant differences in the mean log_10_ CFU/100 cm^2^ among HP1, HP2, HP3, QA1, and QA3 (*P* ≥ 0.05; Figs. 4 & 5).

### The cross-contamination risk presented by disinfectant towelettes varies between *S. aureus and P. aeruginosa*

The surface area wiped and strain type were statistically significant (*P* < 0.0001). Overall, disinfectant towelettes transferred significantly higher log_10_ CFU/100 cm^2^ of *P. aeruginosa* than *S. aureus* to previously uncontaminated surfaces (*P* < 0.05) and there were significant differences among the tested products (*P* < 0.05). For both *S. aureus* and *P. aeruginosa*, surfaces wiped with HP3, QA1, and QA3 had significantly lower log_10_ CFU/100 cm^2^ post-disinfection than HP1 and QA2 (*P* < 0.05). Similarly, for both *S. aureus* and *P. aeruginosa*, the log_10_ CFU/100 cm^2^ detected from the i-zone for HP1 and HP2 were statistically similar after disinfection (*P* ≥ 0.05; Figs. 2 & 4).

### Viable *P. aeruginosa* and *S. aureus* were found on disinfectant towelettes after use

**Fig. 6:**
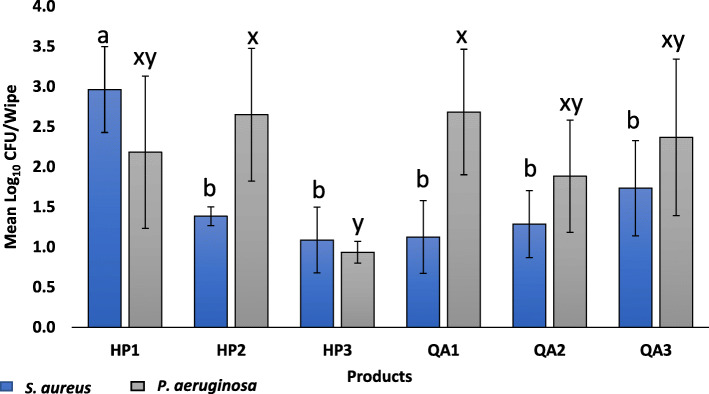
Mean log_10_ CFU remaining on used towelettes post-disinfection with quaternary ammonium alcohol and hydrogen peroxide disinfectant towelettes for *S. aureus* and *P. aeruginosa*. a, ab, b Tukey grouping (mean comparison) for *S. aureus;* x, xy, y Tukey grouping for *P. aeruginosa*. Bars with the same letter are not statistical different

Overall, the bacterial log_10_ CFU remaining on towelettes after disinfection based on the product type used was statistically significant for *S. aureus* (*P* < 0.0053) and *P. aeruginosa* (*P* < 0.0001). For *S. aureus,* log_10_ CFU/wipe ranged from 1.09 ± 0.41 for HP3 to 2.96 ± 0.54 for HP1. Residual *P. aeruginosa* ranged from 0.94 ± 0.13 for HP3 to 2.69 ± 0.78 for QA1 (Fig. 6). When comparing *S. aureus* and *P. aeruginosa*, strain type was significant (*P* = 0.0038, Fig. 6). Post-disinfection, all products had a significantly higher mean log_10_ CFU/ towelette of *P. aeruginosa* than *S. aureus* (*P* < 0.05; Fig. 6). Regardless of strain, HP3 wipes had significantly lower average log_10_ CFU/towelette than HP1, HP2, QA1 and QA3 after use (*P* < 0.05; Fig. 6). The mean log_10_ CFU/wipe for HP3 and QA2 post-disinfection were not significantly different (*P* > 0.05; Fig. 6) and were not different by strain.

Among the wipes used to disinfect surfaces inoculated with *S. aureus*, there were no statistically significant differences among QA1, QA2, or QA3 (*P* ≥ 0.05; Fig. 6). For the HP products, HP1 had significantly higher log_10_ CFU/towelette than HP2 and HP3 (*P* < 0.05; Fig. 6). Similarly, HP1 had significantly higher log_10_ CFU/ towelette than QA1, QA2 and QA3 (*P* < 0.05; Fig. 6). However, there were no statistically significant differences in the log_10_ CFU/ towelette among HP2, HP3, QA1, QA2, and QA3 after use (*P* ≥ 0.05; Fig. 6).

Among the wipes used to disinfect *P. aeruginosa,* there were no significant differences in the mean log_10_ CFU/ towelette among QA1, QA2 and QA3 (*P* > 0.05; Fig. 6) after use. However, for the accelerated HP products, HP3 had the lowest log_10_ CFU/ towelette and was statistically significant in comparison to disinfectant towelettes of HP2 post-disinfection (*P* < 0.05; Fig. 6). There were, however, no significant differences between the average log_10_ CFU/wipe between HP1 and HP3 (*P* > 0.05; Fig. 6). Similarly, there were no statistically relevant differences in the average log_10_ CFU detected on towelettes of HP1, QA2 and QA3 (*P* > 0.05; Fig. 5) and between HP2 and QA1 towelettes post-disinfection *P* > 0.05; Fig. 6).

## Discussion

In this study, we evaluated the cross-contamination risk that may be presented by disinfectant towelettes during and after the wiping process. During the wiping procedure, disinfectant towelettes transferred the test pathogens from the i-zone to uncontaminated surfaces (0.5 m^2^, 1.0 m^2^, 1.5 m^2^ and 2.0 m^2^), with significant differences between the log_10_ CFU/100 cm^2^ transferred by the products tested. We also found that post-disinfection, all used towelettes retained some level of *S. aureus* or *P. aeruginosa,* with some significant differences among the tested products. Overall, HP3, QA1 and QA3 had higher log_10_ reductions than the other products tested.

### Used disinfectant wipes are potential cross-contamination agents for *S. aureus*

Overall, the tested disinfectant towelettes transferred *S. aureus* from the i-zone to previously uncontaminated surfaces. This is similar to findings by Ramm et al. who studied the potential for detergent wipes to transfer *S. aureus* from a contaminated stainless steel surface onto three other surfaces [27]. Specifically, Ramm et al. found that all seven disinfectant towelettes tested cross-contaminated the sterile stainless steel surfaces [27]. The transfer of pathogens to hard nonporous surfaces as demonstrated by our study is particularly important as pathogens are more easily transferred from hard nonporous surfaces to human hands than from porous surfaces [28].

We found no significant differences among the log_10_ CFU/100 cm^2^ remaining on uncontaminated surfaces post-disinfection. In a similar study with methicillin resistant *S. aureus* (MRSA) inoculated on stainless steel discs, Williams et al. did not find significant differences in the log_10_ CFU detected post-disinfection with towelettes [29]. While overall there were no significant differences in the log_10_ CFU/100 cm^2^ of *S. aureus* transferred among all six products we tested, QA1 transferred significantly lower log_10_ CFU/100 cm^2^ to the 1.5 m^2^ zone than all the other products. This difference may be accounted for by the high alcohol content (55% alcohol) of QA1 (Table 1).

### Quaternary ammonium towelettes transfer more *P. aeruginosa* than hydrogen peroxide towelettes

Hydrogen peroxide products transferred significantly less log_10_ CFU/100 cm^2^ to previously uncontaminated surfaces than QA products. This is similar to previous findings by our group where we demonstrated that hydrogen peroxide disinfectant towelettes were more bactericidal against *S. aureus* and *P. aeruginosa* inoculated on 97 mm Formica disc than quaternary alcohol towelettes [18]. This significant difference could be explained by the potential for accelerated HP products to produce hydroxyl free radicals which are generally more bactericidal than quaternary alcohols [20]. Moreover, Edwards et al. reported that the number of bacterial cells transferred from contaminated to uncontaminated surfaces is dependent on the disinfectant active ingredient type loaded unto the towelettes [30]. This further emphasizes the role disinfectant chemistry plays in the cross-contamination levels observed in our study.

Among the QA products, QA1 and QA3 had a significantly higher log_10_ reduction within the i-zone and also presented a significantly lower cross-contamination risk to uncontaminated surfaces than QA2. Although QA1 and QA3 have differences in their alcohol contents (55% for QA1 and 14.3% for QA3), they had very similar quaternary ammonium contents (0.25% for QA1 and 0.233% for QA3). The similar levels of quaternary ammonium compounds between QA1 and QA3 may have been optimal enough to complement and enhance the bactericidal efficacy of the alcohol levels in these products. This is specifically important as in 2015, Gerba reported that the efficacy of quaternary ammonium compounds is dependent on the product formulation [31].

### *P. aeruginosa* is a higher cross-contamination risk from towelettes than *S. aureus*

Disinfectant towelettes transferred significantly higher log_10_ CFU/100 cm^2^ of *P. aeruginosa* than *S. aureus*. The lipopolysaccharides (LPS) in the outer membrane of Gram negative bacteria may serve as a barrier offering reduced permeability to some disinfectants as this has been specifically demonstrated with quaternary ammonium compounds [32]. In a 2002 study that evaluated the transfer rate of Gram positive and Gram negative bacteria from hard nonporous fomites to the hands of workers, it was found that Gram positive bacteria transfer rates were higher [28].

Regardless of strain, the cross-contamination risk presented by disinfectant towelettes was highly product-dependent as HP3, QA1 and QA3 wipes had significantly lower log_10_ CFU/100 cm^2^ post-disinfection than HP1 and QA2. The observed differences may be explained by factors as differences in the wipe material type [33], active ingredient class [17], and differences in the amount of liquid released onto the test surfaces [18] during the wiping process. The disinfectant towelette substrate type has been demonstrated to play a major role in the physical removal of pathogens from test surfaces [34]. In a 2013 study that compared the bactericidal efficacy of different towelette substrate types composed of either cellulose, cotton, microfiber and a blend of cotton and cellulose loaded with silver dihydrogen citrate, it was found that substrates with a mix of cellulose and cotton were more bactericidal [33]. This may be the case as substrates with cellulose/cotton blends may absorb more disinfectant liquids than the other substrate types tested. It is also likely that differences in the amount of liquid dispensed from the towelettes account for differences we observed in this study and as previously reported [18].

### Used disinfectant wipes are potential reservoirs for recontamination after use

After wiping the Formica sheet, all the disinfectant wipes we tested retained some level of *S. aureus* or *P. aeruginosa*. This is similar to findings by Cheng et al. who reported MRSA on disinfectant towelettes after use on bedrails [35]. This suggests that disinfectant towelettes could continue to pose a cross-contamination risk if used on larger surfaces (e.g. two meters square surface used in this study). Moreover, in a 2015 study, Ramm et al. reported that detergent towelettes are designed to efficiently pick up and retain microorganisms but that this did not always occur [27]. This finding is consistent with our study as towelettes retained viable cells after disinfection and while the level of risk varies significantly by product for *Pseudomonas*, there is some level of risk of cross-contaminate for all the wipes studies. An observational study by Williams et al. reported that environmental staff in healthcare facilities used a single disinfectant towelette on at least five different surfaces during routine cleaning and disinfection [36]. This further emphasizes the risk that some disinfectant towelettes present when they retain high levels of common pathogens causing HAI and are used on multiple surfaces by healthcare personnel. Post-disinfection, wipes were found to have significantly higher mean log_10_ CFU of *P. aeruginosa* than *S. aureus*. The rod shape of *P. aeruginosa* [37] may have allowed for a better fit into the perforations on the tested towelettes compared to *S. aureus* cocci [38]. Our study is limited as the impact of the wipe material type was not specifically evaluated. In addition, we did not study the cross-contamination risk towelettes may present in the presence of soil loads.

## Conclusion

Overall, disinfectant towelettes transferred viable CFU of *S. aureus* and *P. aeruginosa* from the point of inoculation to uncontaminated surfaces, while retaining viable bacterial loads post-disinfection. The results of this study underscore the risk of spreading *S. aureus* and *P. aeruginosa* during and after the wiping process by some products. Considering that bacterial biofilms are more prevalent in nature than planktonic bacteria, conducting similar studies against biofilms on environmental surfaces may provide more insights into the possible cross-contamination risk that disinfectant towelettes present. We also recommend conducting similar studies using other Gram negative and Gram positive bacteria relevant in healthcare settings, as well as with mixed cultures to further understand the likely differences in the cross-contamination risk under relevant healthcare scenarios.

## Data Availability

All quantitative data generated or analyzed during this study are included in this published article.

## Abbreviations

ACH: Acute Care Hospital
CFU: Colony Forming Units
EPA: Environmental Protection Agency
HAI: Healthcare Associated Infections
HP: Hydrogen Peroxide
QA: Quaternary ammonium
TSA: Tryptic Soy Agar
